# Evaluating the comprehensive influences of heat treatment and polydimethylsiloxane on integrated performance of bamboo timber

**DOI:** 10.1039/d0ra08713k

**Published:** 2020-12-08

**Authors:** Ying Zhang, Xin Zhang, Youming Yu, Wenbo Che, Xiaochun Zhang, Junfeng Hou

**Affiliations:** School of Engineering, Zhejiang A & F University Hangzhou Zhejiang 311300 P. R. China 20110039@zafu.edu.cn houjunfeng@zafu.edu.cn; Zhejiang Provincial Collaborative Innovation Center for Bamboo Resources and High-efficiency Utilization Hangzhou 311300 P. R.China

## Abstract

The main objective of this work is to analyse the influence of heat treatment with polydimethylsiloxane on integrated performance of bamboo timber. Bamboo timber was heat treated using polydimethylsiloxane as a medium at 120, 150, 180 and 210 °C for 3 h in this study. Results revealed that the equilibrium moisture content (EMC) and linear swelling ratio of heat-treated bamboo specimens was remarkable decreased with increasing heat treatment temperature. The surface contact angle of water on the bamboo specimens was observed to increase with the increasing heat treatment temperature, indicating the reduction of wettability with water. Additionally, the modulus of rupture (MOR) and modulus of elasticity (MOE) was decreased with the increasing heat treatment temperature and lower than that of untreated specimens. Cellulose crystallinity of bamboo specimens was slightly decreased with the increase of heat treatment temperature. TG-DTG results illustrated a reduction in relative content of hemicellulose, and increase in relative content of lignin and cellulose of bamboo specimens with the increase of heat treatment temperature. Presence of the stretching vibration Si–C in Si–CH_3_ indicated the bonding of siloxane to bamboo timber by forming covalent bonds. The colour of the heat-treated bamboo timber was even deepened after heat treatment, endowing the bamboo timber with better surface decoration performance.

## Abstract 2

The main objective of this work is to analyse the influence of heat treatment with polydimethylsiloxane on integrated performance of bamboo timber.

## Graphical abstract

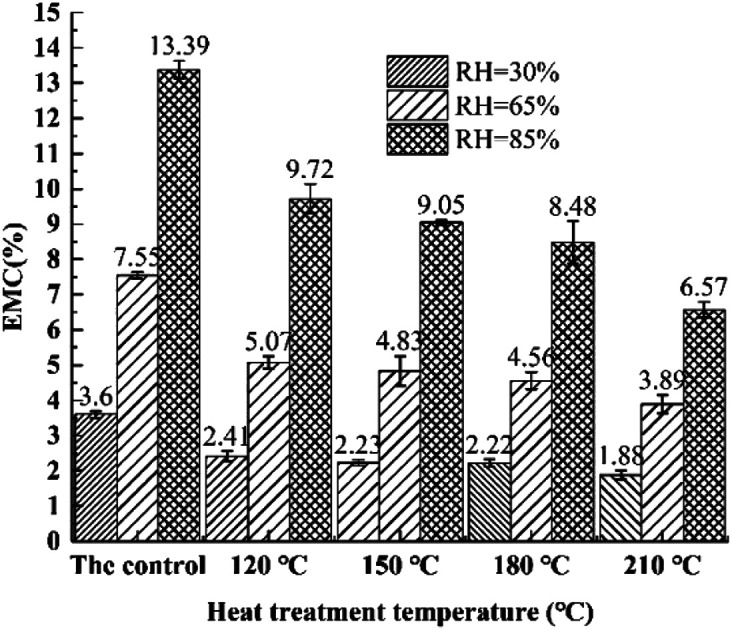

## 1. Introduction

Bamboo is traditionally applied to the fabrication of bamboo plywood, fibreboard and particleboard due to its good reproducibility, high mechanical strength, good processing performance, superior biodegradability and environmental characteristics.^1–4^ Bamboo-based panel has been widely used in manufacturing of furniture, flooring and construction in recent years. However, shortcomings of bamboo such as structural non-uniformity, anisotropy, dimensional instability, shrinkage and moisture swelling, and its high susceptibility to fungal degradation poses a serious threat to the strength and product quality of bamboo products.^5^ Therefore, exploring how to effectively modify bamboo materials to improve the corresponding dimensional stability and surface hydrophobicity is of far-reaching significance.

In view of the shortcoming of poor dimensional stability, shrinkage, moisture swelling, and its high susceptibility to fungal degradation, several treatment methods such as heat treatment,^6–8^ acetylation treatment^9^ and surface finishing with coatings^10^ have been applied so as to improve the dimensional stability and surface hydrophobicity of bamboo materials. Among which, heat treatment is an excellent method of improving integrated performance of bamboo materials.^11–13^ It has been reported the colour difference, weather resistance, dimensional stability, physical and chemical mechanics, durability of bamboo materials was improved by changing the surface structure or chemical composition of materials during thermal modification at various increased temperatures.^13^ Carbonization reaction was also observed to occur to the bamboo timber in a non-oxygen inert environment in a high temperature and high-pressure process. From the previous researches, oil,^14,15^ flue gas,^1^ steam,^16^ acid and alkali^17,18^ was always selected as heat treatment mediums to complete the heat treatment process of bamboo materials. It has been reported that the relative content of hemicellulose in the bamboo timber was generally decreased, and the relative content of cellulose was increased during heat treatment.^19^ The relative content of lignin was also observed to increase, helping to improve the dimensional stability, water repellence and mildew resistance of heat-treated bamboo materials.^14^ A great deal of researches has been performed to analyse the influences of heat treatment temperature, treatment time and treatment medium on physic-chemical performance of bamboo materials.^15,20,21^ Meanwhile, polydimethylsiloxane has been played a more and more important role in the preparation of heat-resistant and moisture-proof fillers owing to its excellent moisture resistance, well light transmission and excellent chemical stability performance. Analysis on the influences of high temperature treatment with oil as medium on the starch content and mold-resistant property revealed that the starch content of heat-treated bamboo specimen using oil as medium was lower than the untreated bamboo, and the mold resistance property of bamboo was obvious improved after heat treatment with oil as medium.^22^ It has been revealed that colour of bamboo timber heat-treated using polydimethylsiloxane as medium was even deepened with the increasing heat treatment time.^23^ However, no research effort has been found which studied the physicochemical properties of bamboo timber heat-treated with polydimethylsiloxane as medium. Research effort is needed to explore the comprehensive influences of high temperature treatment & polydimethylsiloxane on physicochemical properties of bamboo timber.

This study aimed to explore the effects of heat treatment using polydimethylsiloxane as medium on comprehensive performance of bamboo timber. Effect of heat treatment temperature on physical, mechanical, chemical and colour variety of bamboo materials was investigated. A better bamboo modification process is obtained to enhance strength, dimensional stability, surface decoration performance and waterproof property of bamboo materials, with the hope of providing technical basis for promoting the efficient use of bamboo materials.

## 2. Materials and methods

### 2.1 Preparation of bamboo specimens for heat treatment

Five-year-old bamboo (*Phyllostachys heterocycle*) culms were purchased from Lin'an district in Hangzhou city of Zhejiang province, China. The fresh bamboo stalks were sawn into strips with the dimension of 250 mm (length, *L*) ×20 mm (width, *W*) × 5 mm (thickness, *T*). The fresh bamboo strips were air-dried to the moisture content (MC) of 12 ± 0.5% before heat treatment. All of the prepared bamboo strips were evenly divided into 5 groups with 12 specimens in every group.

### 2.2 Heat treatment with polydimethylsiloxane of bamboo materials

Heat treatment for 4 groups of bamboo strips with polydimethylsiloxane (201 type, viscosity 1000 MPa s (250 °C), Shanghai TITAN Technology Co. Ltd) as medium were performed in a homemade oil bath dip tank. The samples were put into cold polydimethylsiloxane and heated by a homemade oil bath dip tank until the temperature of polydimethylsiloxane was increased to the preset heat treatment. The preset heat treatment temperature was 120, 150, 180 and 210 °C, respectively. The holding time of treatment process was 3 h. The heat-treated specimens were taking out from hot polydimethylsiloxane and cooled down to room temperature. The bamboo strips were dried before and after heat treatment with polydimethylsiloxane to ensure the fully absorption of polydimethylsiloxane by bamboo strips.

### 2.3 Measurement of physical and mechanical properties

The heat-treated and untreated bamboo specimens were cut into a size of 20 mm (*L*) × 20 mm (*W*) × 5 mm (*T*) to investigate EMC and swelling ratio. 12 replicate specimens for each type were measured for EMC and swelling ratio results analysis. The presented values for EMC and swelling ratio was the average value. The bamboo specimens were processed in an atmosphere environment with a temperature of 17.5 °C and relative humidity (RH) of 69.6%, for thirty days and weighed. And then the whole specimens were dried at 103 ± 2 °C in a drying oven (DKN611 Yamato Scientific Co. LTD. Tokyo Japan) to analyse weight and parameters of dimension in length, width and thickness orientation at oven-dry state.

All of the bamboo specimens were divided into 3 groups. Dimension and moisture content (MC) of specimens were investigated at 20 °C/30% RH, 20 °C/65% RH and 20 °C/85% RH condition in a constant temperature–humidity chamber (LHU-113 ESPEC CORP Tokyo Japan), and the numerical values of individual specimens were measured periodically until constant reading reached. Air-dried density was calculated in accordance with the specimen weight in g and dimensional parameters in cm at an equilibrium state with RH of 65% at 20 °C.

Bamboo specimens heat-treated and untreated were cut into a size of 120 mm (*L*) × 20 mm (*W*) × 5 mm (*T*) for determination of modulus of elasticity (MOE) and modulus of rupture (MOR) according to GB/T 15780-1995: Testing methods for physical and mechanical properties of bamboos.^24^ Prior to the mechanical property testing, the tested specimens were processed to the equilibrium moisture content (EMC) of 12% at a controlled environment of 20 °C/65% RH in a high-low humidity alternating test box (EL-10KA, Espec Corporation, USA). A total of 12 bamboo specimens for bending tests (MOR and MOE) were measured for the final results analysis. The presented values for MOR and MOE tests were the average value.

### 2.4 Analysis of contact angle of water on bamboo specimen

Bamboo specimens in the heat-treated and control group were cut into a size of 60 mm (*L*) × 20 mm (*W*) × 5 mm (*T*) for determination of contact angle of water. Prior to the testing, the tested specimens were processed to EMC (12%) at a preset environment (20 °C/65% RH) in a high-low humidity alternating test box (EL-10KA, Espec Corporation, USA). Surface contact angle of water on bamboo specimen was investigated by automatic contact angle measuring instrument (TBU100, Dataphysics Instrument Co. Ltd. German). Six replicate specimens for each type were measured for the analysis of contact angle of water on bamboo specimen. Five points for each specimen was tested and the presented values were the mean value of 30 replicate data.

### 2.5 Fourier transform infrared spectroscopy (FTIR) analysis

Fourier transform infrared spectroscopy (FTIR) analysis was conducted using a spectroscopy (Nicolet 6700, Nicolet Co. Ltd. USA) at frequencies ranging from 400 cm^−1^ to 4000 cm^−1^. The compressed tablet for FTIR test was prepared by mixing 1 mg of bamboo powders at oven-dry state selected by an 80–120-mesh sieve with 99 mg of potassium bromide (KBr).^5^

### 2.6 (X-ray diffraction) XRD analysis

The test was performed using an X-ray diffraction analyzer (XRD-6000, Shimadzu, Japan) with Cu radiation (40 kV and 30 mA). Prior to the XRD test, 0.2 g of bamboo powders at oven-dry state were selected by an 80–120-mesh sieve. The scanning 2*θ* ranged from 5° to 90° at a step of 2° min^−1^.

### 2.7 TG-DTG measurement

For the simultaneous thermal analysis (STA 409 C, NETZSCH Company, German), 10 mg of bamboo powders was placed in an Al_2_O_3_ crucible and measured by heating from 20 °C to 800 °C with a heating rate of 10 °C min^−1^ in nitrogen atmosphere. TG (Thermogravimetry) and DTG (Derivative Thermogravimetry) curves were recorded in the whole test process.

### 2.8 Color measurement

Surface color of heat treated and untreated specimens was measured by DC-P3 new type automatic colorimeter in accordance with the CIE L*a*b* color system with a diameter of measured circle of 10 mm and light source of Standard Illuminant D65. 20 replicate specimens for each type were measured for colour measurement results analysis. The presented values for colour difference was the average value. Among which, L* is the parameter of lightness, a* is the chroma from green to red, and b* is the chroma from blue to yellow.^25^ The color difference can be calculated by using eqn (1):^25^1
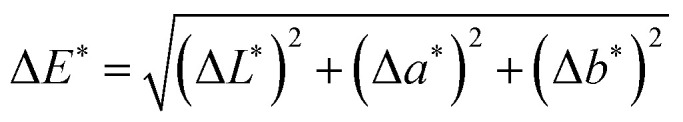
where, ΔE* is the color difference, ΔL* is the lightness difference, and Δa* and Δb* are the chroma differences. Δ means the differences between the initial and final parameters of the specimens after heat treatment.

## 3. Results and discussion

### 3.1 Performance analysis of physics and mechanics

**Fig. 1:**
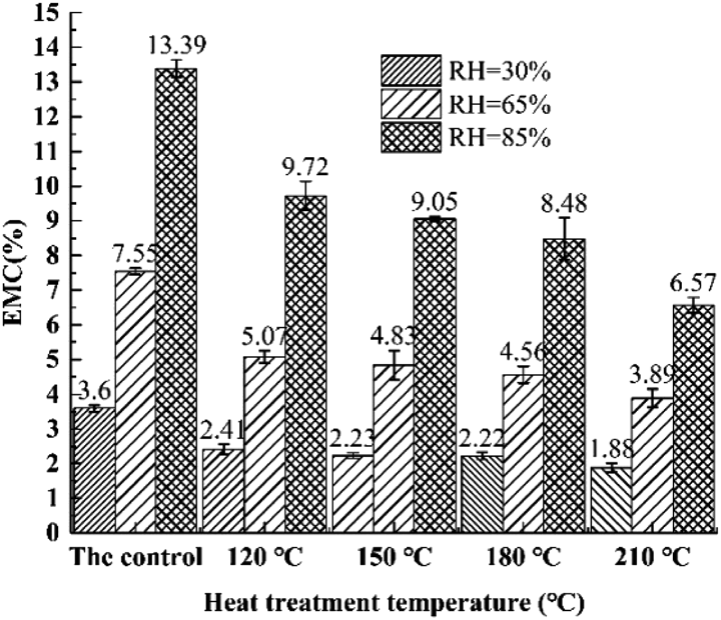
Effects of heat treatment temperature on EMC of bamboo specimens.

Fig. 1 presents the influence of heat treatment temperature on EMC of bamboo specimen. A gradual increase was observed to the EMC of heat-treated bamboo specimen with increasing environment humidity. Furthermore, EMC of treated specimens was obvious reduced with the increase of heat treatment temperature.^21^ Bamboo timber is mainly composed of cellulose, hemicellulose, and lignin. The hygroscopicity of bamboo is related to free hydroxyl groups presented on cellulose and hemicellulose.^26^ Migration rate of bonding water from bamboo surface was increased with increasing temperature. Among which, the gradually removal of free hydroxyl group is the main reason why the EMC of bamboo specimens was declined with the rising heat treatment temperature.

Influences of heat treatment temperature on linear swelling ratio of bamboo specimens are illustrated in Table 1. It was indicated that linear swelling rate of specimens were effectively reduced after high temperature treatment with polydimethylsiloxane as medium. Compared with the untreated specimens, tangential swelling ratio of specimens after treatment at 120, 150, 180 and 210 °C was decreased by 30.85%, 36.17%, 52.13%, 59.57%, respectively at 20 °C and 30% RH. Swelling ratio in radial orientation was decreased by 7.69%, 17.86%, 28.57%, and 40.00%, respectively. And the longitudinal swelling ratio was decreased by 9.38%, 15.63%, 21.88%, and 52.38%, respectively. It was indicated that an obvious increase in tangential and radial swelling ratio was observed on account of anisotropy and structure of bamboo itself.^4,27,28^ However, the corresponding values of swelling ratio along longitudinal orientation was not. The linear swelling ratio was observed to decline with the increase of heat treatment temperature. Therefore, dimensional stability of bamboo specimens was effectively improved with high temperature treatment using polydimethylsiloxane as medium. And the higher the temperature is, the better dimensional stability performance of the tested specimens is obtained.

**Table 1:** Effects of heat treatment temperature on swelling ratio of bamboo specimens

Heat treatment temperature/°C	RH = 30%			RH = 65%			RH = 85%		
	*T*	*R*	*L*	*T*	*R*	*L*	*T*	*R*	*L*
The control	0.94 ± 0.13	1.40 ± 0.21	0.32 ± 0.04	1.32 ± 0.15	1.46 ± 0.25	0.33 ± 0.05	2.72 ± 0.35	2.49 ± 0.42	0.40 ± 0.04
120	0.65 ± 0.09	1.30 ± 0.18	0.29 ± 0.02	0.86 ± 0.09	1.38 ± 0.30	0.31 ± 0.04	2.32 ± 0.27	1.87 ± 0.23	0.38 ± 0.05
150	0.60 ± 0.11	1.15 ± 0.09	0.27 ± 0.15	0.73 ± 0.06	1.18 ± 0.19	0.30 ± 0.01	2.19 ± 0.29	1.76 ± 0.15	0.33 ± 0.02
180	0.45 ± 0.06	1.00 ± 0.11	0.26 ± 0.02	0.61 ± 0.05	1.04 ± 0.13	026 ± 0.02	1.32 ± 0.33	1.64 ± 0.18	0.32 ± 0.04
210	0.38 ± 0.02	0.84 ± 0.07	0.22 ± 0.01	0.48 ± 0.04	0.86 ± 0.10	0.22 ± 0.03	0.97 ± 0.14	1.57 ± 0.12	0.28 ± 0.05

**Fig. 2:**
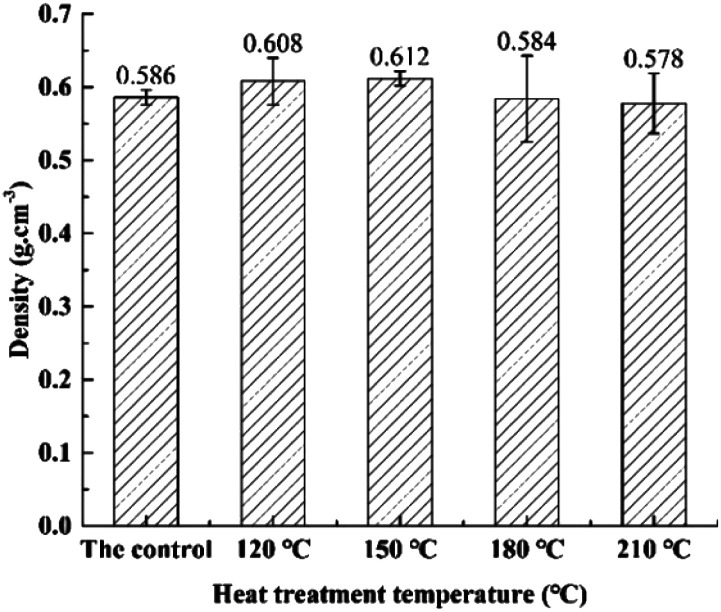
Effects of heat treatment temperature on density of bamboo specimens.

Fig. 2 presents the influence of heat treatment temperature on density of bamboo specimen. Obviously, density of heat-treated specimens was increased with the increase of heat treatment temperature to 150 °C, and declined with the further increase of heat treatment temperature. It can be surmised that density change of tested specimen was obvious influenced by the synergism of polydimethylsiloxane and high temperature treatment decomposition. Effect of polydimethylsiloxane is dominant within the temperature range of 120–150 °C. Impregnation of bamboo specimen cells with polydimethylsiloxane leading to the increase of bamboo specimen weight and further resulted in the slightly increase of density during heat treatment.^6^ It was also noted that violent pyrolysis was observed to generate to bamboo materials, leading to the overall decline of bamboo density with the further increase of temperature from 150 to 210 °C.^29,30^

**Fig. 3:**
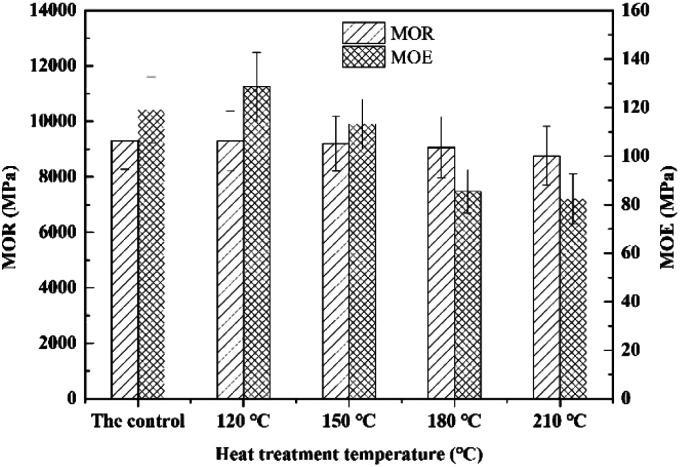
Effects of heat treatment temperature on MOR and MOE of bamboo specimens.

Fig. 3 presents the effects of heat treatment temperature on modulus of rupture (MOR) and modulus of elasticity (MOE) of bamboo specimen. As illustrated in the figure, MOR of specimens was decreased continuously with the increasing heat treatment temperature. With the increasing of heat treatment temperature to 120, 150, 180 and 210 °C, the corresponding reduction was 0.12%, 1.07%, 2.63% and 5.79% in comparison with untreated specimens. Compared with the untreated specimens, an increase of 8.07% was generated to MOR of bamboo specimens heat-treated at 120 °C. Meanwhile, it was only slightly reduced by 4.86% with the further increasing of heat treatment temperature to 150 °C. And the corresponding decrement was 28.24% and 30.71% with the further increase of heat treatment temperature to 180 and 210 °C. This is due to the gradually undergo thermal decomposition of main chemical component namely hemicellulose, lignin and cellulose in the specimens according to their thermal stability with increasing heat treatment temperature and cause a certain mass loss of bamboo specimen, further result in the reduction in mechanical properties during heat treatment.^31,32^ It has been observed that a drastic degradation was generated in bamboo specimen with the further increasing of heat treatment temperature and led to the obvious effect on mechanical property of bamboo specimens. Therefore, an increase followed by decline in mechanical property of bamboo was generated. And the reduction was observed to increase with rising heat treatment temperature from 150 to 210 °C.

A small decrement of 0.12% in MOR of heat-treated bamboo specimens was generated in comparison with the untreated specimens. This is mainly because of a slightly pyrolysis of hemicellulose at the heat treatment temperature 120 °C, while the chemical components of cellulose & lignin in specimens was not changed. Furthermore, MOE was increased to the maximum value with an increase of 8.07% at 120 °C. It has been discovered that variety of static bending strength was mainly dependent on the assimilation of oil, the thermal decomposition of bamboo components and evaporation of moisture during heat treatment with oil as medium. Absorption of oil plays a decisive role during the low temperature stage of heat treatment for bamboo specimens, and results in the improvement of mechanical property of bamboo specimen in some extent. Additionally, decreasing of moisture content (MC) caused by water evaporation resulting in the increase of static bending strength of bamboo materials with its MC lower than FSP. Crystallization was also observed to generate to xylan and mannan in hemicellulose in a relatively low temperature stage of heat treatment for bamboo specimens. Therefore, increasing in the crystallinity of heat-treated bamboo specimens leading to the increases of mechanical property.^28^ While, thermal decomposition of bamboo materials is intensified, and the static bending strength of bamboo is observed to decline with the further increasing heat treatment temperature during high temperature stage. It also has been discovered by Cheng^6^ that static bending strength of bamboo specimen is mainly dependent on the thermal decomposition of bamboo components and silicon oil absorbed during heat treatment of bamboo using silicon oil as medium. The latter plays a more important role in the lower temperature stage, leading to the generation of an increase in mechanical strength of bamboo specimens. Besides, thermal decomposition is intensified, and causing a decreasing of static bending strength for bamboo specimens with the further increase of heat treatment temperature.

### 3.2 Analysis of contact angle of water on bamboo specimen

**Fig. 4:**
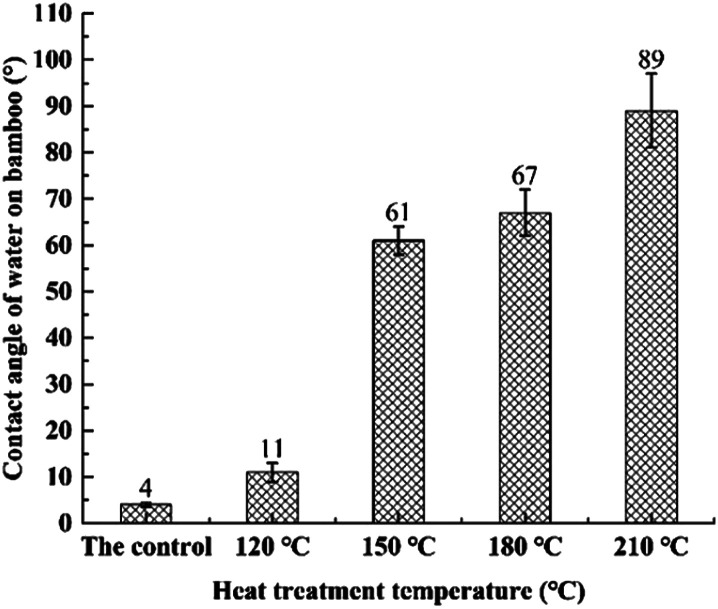
Effects of heat treatment temperature on contact angles of bamboo specimens.

Fig. 4 presents the contact angle of water on heat-treated and untreated bamboo specimens. As illustrated in Fig. 4, contact angle (4°) of water on the untreated specimens was extremely small because of its great wettability with a great deal of free hydroxyl groups located on the surface of bamboo specimens. A gradual increase in the contact angle of water on heat-treated bamboo specimens was observed to generate with the increase of heat treatment temperature. And wettability with water of heat-treated bamboo specimens was decreased accordingly. This is because of an obvious decrease in the quantity of hydrophilic free hydroxyl groups in hemicellulose and cellulose with the increase of heat treatment temperature.^16^ Additionally, wettability performance of heat-treated bamboo specimens was also decreased owing to the excellent hydrophobicity of polydimethylsiloxane.^27^ Meanwhile, the differences in contact angles of water on bamboo specimens were also due to change of porosity. Water was fast absorbed into highly porous untreated bamboo specimens what also contributes to low contact angles observed and once heat treated with polydimethylsiloxane as medium, bamboo timber becomes less porous and, absorption of polydimethylsiloxane is lower and the observed contact angles is higher.

### 3.3 FTIR analysis

**Fig. 5:**
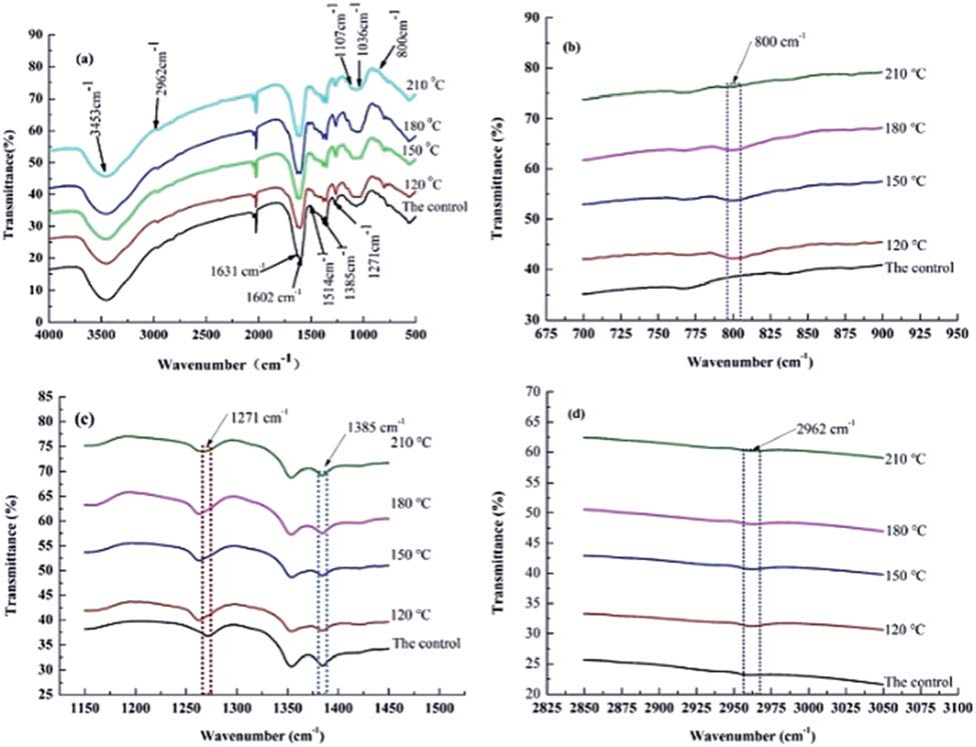
FTIR curves of bamboo specimens heat-treated at different temperatures: (a) FTIR curves of bamboo specimens; (b) 800 cm^−1^; (c) 1271 cm^−1^; (d) 2962 cm^−1^.

FTIR curves of treated and untreated bamboo specimens are illustrated in Fig. 5. Hemicellulose, lignin and cellulose in bamboo will undergo different degrees of thermal degradation during the heat treatment stage in accordance with their own thermal stability.^33,34^ As can be seen in Fig. 5, an obvious stretching vibration of –OH was generated at 3453 cm^−1^ (Fig. 5(a)), and a gradual reduction was observed to the absorption peak intensity with the rise of heat treatment temperature. Mainly bonding water was removed from bamboo specimens with the temperature lower than 100 °C. With the further increase of heat treatment temperature, dehydration polycondensation reaction was observed to occur among the free hydroxyl groups on cellulose molecular chain of bamboo specimens and lead to the forming of ether bond. Amount of free hydroxyl groups in non-crystalline regions of cellulose & hemicellulose was remarkably reduced, leading to an obvious reduction in the intensity of –OH absorption peak. Additionally, a new absorption peak was observed to appear at 2962 cm^−1^ (Fig. 5(d)) in the FTIR curves of heat-treated bamboo specimens, showing the generation of stretching vibration of Si–C bond in Si–CH_3_ of polydimethylsiloxane in the heat treatment process with polydimethylsiloxane as medium. Stretching vibration of C=O at 1735 cm^−1^ (Fig. 5(a)) was significantly reduced with the increases of heat treatment temperature, this is most likely due to acetylation in hemicellulose.^32^ The peak was not observed to generate in FTIR curve of untreated specimens as illustrated in Fig. 5(a). Intensity decreasing in the absorption peak at 1632 cm^−1^ was caused by the stretching vibration of conjugated carbonyl group C=O, indicating the hydroxyl group and conjugated carbonyl group were destroyed after heat treatment with polydimethylsiloxane as medium, and the obvious reduction of adsorbed water content in heat-treated bamboo specimen. Absorption peak at 1602 and 1514 cm^−1^ indicates the stretching vibration of carbon skeleton in lignin benzene ring. A gradual increase was observed to the absorption peak intensity with the increase of heat treatment temperature, indicating thermal decomposition of hemicellulose in bamboo specimens and increasing of lignin content in bamboo specimen after heat treatment. Intensity decreasing in absorption peak at 1385 cm^−1^ (Fig. 5(c)) caused by bending vibration of C–H in cellulose & hemicellulose indicating the thermal decomposition of cellulose in bamboo specimens. An absorption peak was observed to generate at 1271 cm^−1^ (Fig. 5(c)) owing to a stretching vibration of lilac base ring and C–O in lignin and xylan.^35^ The absorption peak intensity was increased with the increasing of heat treatment temperature to 120 °C, and then reduced with the further increasing heat treatment temperature. This may attribute to the thermal decomposition of hemicellulose and increasing in the relative content of lignin in a lower temperature stage. Thermal degradation was occurred to lignin in a large degree with the further increase of heat treatment temperature. Decreasing in the relative content of lignin resulted in the reduction of absorption peak value. Absorption peak at 1107 and 1036 cm^−1^ (Fig. 5(a)) mainly illustrate the presence of stretching vibration of C–O–C and C–O bond in cellulose. And generation of absorption peak at 800 cm^−1^ was caused by Si–O–Si bond.^6^ A new band at 2962 (Fig. 5(d)) and 800 cm^−1^ (Fig. 5(b)) observed in heat treatment bamboo specimens proved the forming of stretching vibration Si–C in Si–CH_3_ of polydimethylsiloxane, indicating the bonding of siloxane to bamboo timber by forming covalent bonds.

### 3.4 XRD analysis

**Fig. 6:**
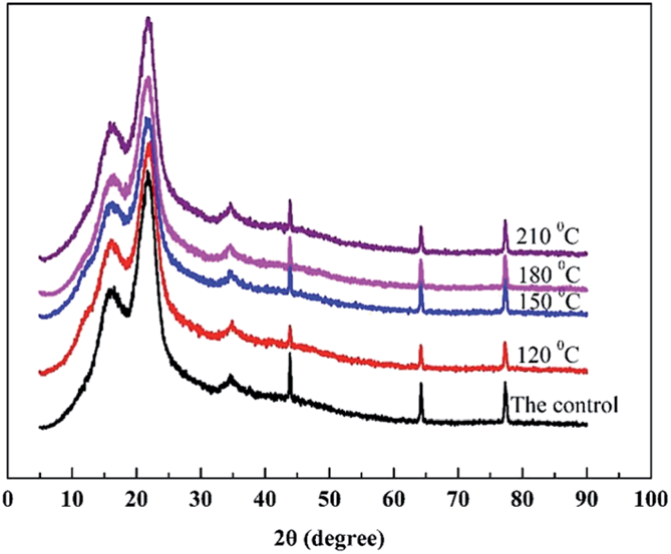
XRD curves of bamboo specimens before and after heat treatment.

XRD curves of treated and untreated bamboo specimens are presented in Fig. 6. Cellulose crystallinity of treated and untreated bamboo specimens at different heat treatment temperatures was generally determined by Segal method.^11^ It was found that the diffraction peaks of 002 plane of bamboo specimens were concentrated around 22°, indicating the application of heat treatment using polydimethylsiloxane as medium does not change the interplanar spacing of cellulose crystal region in bamboo specimens. However, the diffraction peak size was observed to decline with the increase of heat treatment temperature slightly. Compared with the untreated specimens (50.34%), crystallinity of cellulose of heat-treated bamboo specimens at 120, 150, 180 and 210 °C was decreased by 2.74%, 7.47%, 1.63% and 1.25%, respectively. This is mainly due to the generation of acetic acid caused by hemicellulose hydrolysis in heat-treated bamboo specimens in the heat treatment process, creating an acidic environment, which causes the micro fibrils of cellulose to degrade to a certain extent, thereby causing a reduction in cellulose crystallinity.^17^ It can also be seen that heat treatment with polydimethylsiloxane as medium has little effect on cellulose crystallinity of bamboo materials, which may be ascribe to the competitive decomposition of amorphous cellulose and crystalline cellulose.

### 3.5 TG-DTG analysis

**Fig. 7:**
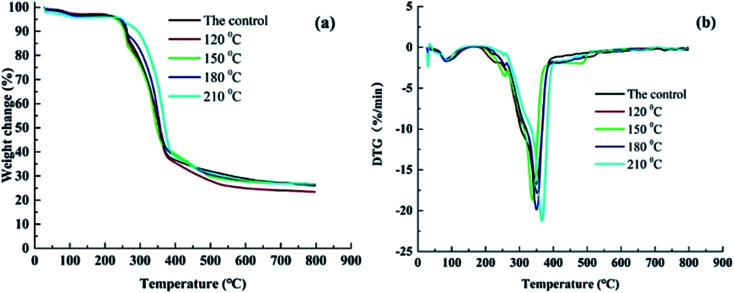
(a) Weight change curves of heat-treated bamboo specimens at different temperatures. (b) DTG curves of heat-treated bamboo specimens at different temperatures.

Fig. 7 shows the thermogravimetric curves of heat-treated bamboo. The pyrolysis process of bamboo is mainly divided into three stages in accordance with the heat treatment temperature.^19,32^ For the first stage with temperature from 37 to 178 °C, the heat transferred to bamboo by contact heat transfer method was absorbed by bonding water in bamboo, and resulted in the vaporization of bonding water. The evaporation of bonding water plays a dominant role with the temperature lower than 100 °C. Content of bond water in bamboo specimens started to decline with the further increasing temperature. However, chemical components in bamboo materials is basically unchanged in the 1^st^ stage. The second stage is the rapid pyrolysis stage with a temperature range of 178–390 °C. This pyrolysis stage usually divided into 2 steps: the corresponding temperature range is 178–264 °C and 264–390 °C.^18^ A drastic thermal degradation was occurred to easy decomposition component of hemicellulose in bamboo materials in the temperature ranging from 178 °C to 264 °C, and resulted in the quickly loss of bamboo weight. A representative peak appeared at about 260 °C in the DTG curves conformed this conclusion. The most intense pyrolysis of bamboo specimens was generated in the temperature ranging from 264 °C to 390 °C, and the largest weight loss ratio was obtained at about 350 °C.^32^ The presence of intense thermal in this stage was confirmed as the thermal degradation of cellulose & lignin in heat-treated specimens. And the biggest peak in DTG curves was observe to generate at about 350 °C as illustrated in Fig. 7(b). And the peak was increased with the rise of heat treatment temperature. The hydroxyl and carbon–oxygen combination in cellulose & hemicellulose of the tested specimens were observed to be seriously damaged in this stage. The third stage is a slow pyrolysis stage with temperature greater than 390 °C, and it is observed to appear as a tailing peak in DTG curves. Weight loss rate of the tested specimens was obviously decreased in comparison with the second stage, and weight loss curves of bamboo specimens tend to be gentle as illustrated in Fig. 7. A re-degradation was observed to occur to the incompletely pyrolysis components of bamboo in this stage. Heat treatment temperature applied to this study was 120, 150, 180 and 210 °C, and the reaction only appeared in the thermal degradation within the temperature lower than 390 °C (*i.e.* the first and second stage of bamboo thermal degradation). Pyrolysis peak of bamboo specimen in the 1^st^ step during the rapid pyrolysis of bamboo was decreased and pyrolysis peak in the 2^nd^ step was increased gradually with the increasing heat treatment temperature. This is because of obvious pyrolysis of specimens under the comprehensive effects of high temperature heat treatment and polydimethylsiloxane, resulting in an obvious variety for the distribution of various chemical components. Relative content of hemicellulose in treated specimens was observed to decline, and that of the lignin and cellulose was increased.^14,18^

### 3.6 Color changes analysis

**Fig. 8:**
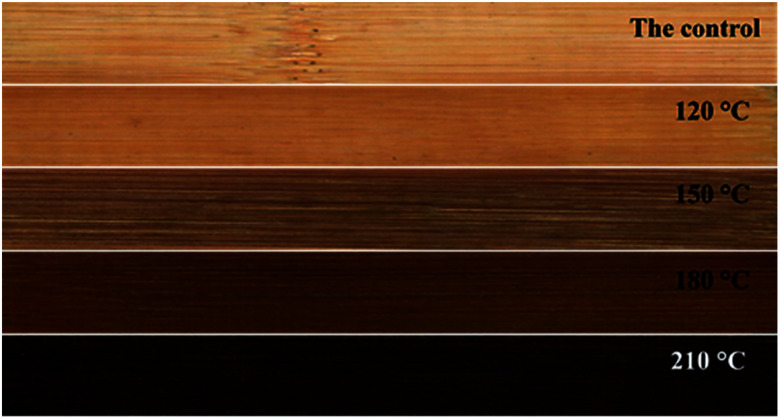
Effect of heat treatment temperature on surface color of bamboo specimen.

Surface color of bamboo specimens before and after heat treatment with polydimethylsiloxane as medium is presented in Fig. 8. Colour changes of heat-treated bamboo specimens at different temperatures is illustrated in Table 2. A reduction was generated to L* of heat-treated specimens, indicating darken of bamboo materials after 3 h heat treatment using polydimethylsiloxane as medium. The corresponding changes in values of L* was −7.08, −22.55, −37.1, −62.26 at 120, 150, 180 and 210 °C compared with the specimens in control group. The same conclusion is consistent with the results presented in Fig. 8. As illustrated in Table 2, reduction of lightness of heat-treated bamboo specimens was increased from 10.68 to 93.94 with the increase of heat treatment temperature from 120 to 210 °C. Additionally, green-red (a*) and yellow-blue (b*) colour coordinates were also changed significantly at different heat treatment temperatures. The negative values of Δa* indicates an obvious tendency for surface of the bamboo specimens to become greenish.^33,36^ Δa* of bamboo specimens treated at 120, 150, 180 and 210 °C was −0.44, −5.97, −6.98 and −1.07, respectively, indicating that the heat-treated bamboo has a tendency to become greenish in varying degrees. Additionally, the values in Δb* of specimens treated at 120, 150, 180 and 210 °C was 173.98, 266.93, 490.82 and 2570.21, respectively, indicating a tendency of the surface of treated specimens to become yellow. Color change of Δb* for the tested specimens was greater than that of the values of Δa*, indicating the much greater influence of high temperature treatment using polydimethylsiloxane on the yellowing degree of the tested specimens. Total color difference (ΔE*) is a comprehensive indicator of color change for treated and untreated bamboo specimens. It can be seen from Table 2 that a gradual increase in ΔE* was observed with the increase of heat treatment temperature. Color changes generated to the tested specimens at different heat treatment temperatures was caused by pyrolysis of hemicellulose & polysaccharides to some extend.^35^ Condensation reaction occurs to the lignin side chain during heat treatment, results in the generation of new β–β and β-5 condensation structures and new conjugated double bonds, and the conjugated system is lengthened, causing the moving of UV absorption band to the visible light region and deepening of the bamboo color.^23^ It is also worth noting that the abnormal change of Δa* indicates colour changes of heat-treated bamboo specimens may be influenced by polydimethylsiloxane absorption in the heat treatment process with polydimethylsiloxane as medium.

**Table 2:** Effects of heat treatment temperature on color changes of bamboo specimens

Heat treatment temperature/°C	Color in CLE L*a*b* system			Color difference				Color change (%)		
	L*	a*	b*	ΔL*	Δa*	Δb*	ΔE*	ΔL*	Δa*	Δb*
The control	66.28 ± 0.49	35.05 ± 0.70	−808.86 ± 190.70							
120	59.20 ± 0.16	34.61 ± 0.61	−634.88 ± 149.87	−7.08 ± 0.37	−0.44 ± 1.27	173.98 ± 43.89	174.12 ± 43.79	−10.68 ± 0.48	−1.26 ± 0.35	−21.51 ± 2.00
150	43.73 ± 1.12	29.08 ± 1.96	−541.93 ± 101.88	−22.55 ± 1.58	−5.97 ± 2.49	266.93 ± 88.68	267.94 ± 88.68	−34.02 ± 2.12	−17.03 ± 2.11	−33.00 ± 2.94
180	29.18 ± 26	28.07 ± 1.12	−318.04 ± 74.54	−37.10 ± 0.68	−6.98 ± 1.71	490.82 ± 120.35	492.27 ± 120.35	−55.97 ± 0.65	−19.91 ± 4.68	−60.68 ± 1.85
210	4.02 ± 0.95	33.98 ± 5.88	1761.36 ± 447.65	−62.26 ± 1.14	−1.07 ± 6.55	2570.21 ± 338.02	2570.97 ± 327.00	−93.93 ± 1.44	−3.05 ± 0.44	−317.76 ± 57.69

## 4. Conclusions

This work systematically investigates the effects of heat treatment with polydimethylsiloxane as medium on comprehensive performance of bamboo timber at the heat treatment temperature ranging from 120 to 210 °C. Results of physical property revealed that EMC and swelling ratio of heat-treated bamboo specimens was decreased with the rise of heat treatment temperature. Increasing in the surface contact angle of water on heat-treated specimens indicating the reduction of wettability with water for bamboo specimen with the increase of heat treatment temperature. Results of mechanical property revealed that the maximum value of MOR was obtained at 120 °C. Additionally, MOE was increased with the increasing heat treatment temperature and lower than that of the untreated bamboo specimens. XRD results showed a slightly decreasing in cellulose crystallinity of heat-treated bamboo specimens with the increasing heat treatment temperature. A new band at 2962 and 800 cm^−1^ found in the heat-treated bamboo specimens confirmed the presence of stretching vibration Si–C in Si–CH_3_ of polydimethylsiloxane, indicating the bonding of siloxane to bamboo timber by forming covalent bonds. Results of TG-DTG analysis also illustrated a reduction in the relative content of hemicellulose, and increase in the relative content of lignin and cellulose of bamboo specimens with the increase of heat treatment temperature. Colour of heat-treated bamboo specimens is even deepened in the whole thickness and length direction, which endows bamboo materials with well surface decoration performance. This work offers a better bamboo modification process to improve mechanical strength, dimensional stability, surface decoration performance, and waterproof performance of bamboo timber, with the hope of providing technical basis for improving the application of bamboo timber.

## Conflicts of interest

There are no conflicts to declare.

## Supplementary Material
